# Glycolysis regulates the expansion of myeloid-derived suppressor cells in tumor-bearing hosts through prevention of ROS-mediated apoptosis

**DOI:** 10.1038/cddis.2017.192

**Published:** 2017-05-11

**Authors:** Shiou-Ling Jian, Wei-Wei Chen, Yu-Chia Su, Yu-Wen Su, Tsung-Hsien Chuang, Shu-Ching Hsu, Li-Rung Huang

**Affiliations:** 1 Institute of Molecular and Genomic Medicine, National Health Research Institutes, Miaoli, Taiwan; 2 National Laboratory Animal Center, National Applied Research Laboratories, Taipei, Taiwan; 3 Immunology Research Center, National Health Research Institutes, Miaoli, Taiwan; 4 National Institute of Infectious Diseases and Vaccinology, National Health Research Institutes, Miaoli, Taiwan

## Abstract

Immunotherapy aiming to rescue or boost antitumor immunity is an emerging strategy for treatment of cancers. The efficacy of immunotherapy is strongly controlled by the immunological milieu of cancer patients. Myeloid-derived suppressor cells (MDSCs) are heterogeneous immature myeloid cell populations with immunosuppressive functions accumulating in individuals during tumor progression. The signaling mechanisms of MDSC activation have been well studied. However, there is little known about the metabolic status of MDSCs and the physiological role of their metabolic reprogramming. In this study, we discovered that myeloid cells upregulated their glycolytic genes when encountered with tumor-derived factors. MDSCs exhibited higher glycolytic rate than their normal cell compartment did, which contributed to the accumulation of the MDSCs in tumor-bearing hosts. Upregulation of glycolysis prevented excess reactive oxygen species (ROS) production by MDSCs, which protected MDSCs from apoptosis. Most importantly, we identified the glycolytic metabolite, phosphoenolpyruvate (PEP), as a vital antioxidant agent able to prevent excess ROS production and therefore contributed to the survival of MDSCs. These findings suggest that glycolytic metabolites have important roles in the modulation of fitness of MDSCs and could be potential targets for anti-MDSC strategy. Targeting MDSCs with analogs of specific glycolytic metabolites, for example, 2-phosphoglycerate or PEP may diminish the accumulation of MDSCs and reverse the immunosuppressive milieu in tumor-bearing individuals.

Immunotherapy aiming to promote tumor-specific immunity in cancer patients for treatment of cancer is a developing field. Cancer vaccine alone failed to induce a complete clinical response in most of the cases. Whereas immune checkpoint inhibitors blocking PD-1 and CTLA-4 signaling have achieved a great success in the treatment of cancer patients,^1, 2^ immune checkpoints are not the only mechanisms for T-cell suppression in the tumor microenvironment. Immunosuppressive cell populations harbor inhibitory mechanisms, for example, arginase 1, iNOS and NAPDH oxidase to induce T-cell proliferative arrest and to inhibit T-cell activation.^3^ Thus, using cancer vaccines to induce tumor-specific T-cell responses in combination with strategies to target immunosuppressive cell populations in cancer patients can be a preferable scheme for the treatment of malignancies.^4^

Myeloid-derived suppressor cells (MDSCs) are an immature myeloid cell (IMC) population, which appear during tumor progression and chronic inflammation and harbor immunosuppression functions able to impair activities of T-cell, NK cells and dendritic cells. MDSCs can be classified into monocytic (CD11b^+^Ly6C^high^Ly6G^−^) and granulocytic MDSCs (CD11b^+^Ly6C^int^Ly6G^high^) based on their nuclear morphology and surface markers.^3^ In tumor-bearing individuals, IMC populations in bone marrow could respond to tumor-derived factors and proliferate through activation of JAK protein family and STAT3 signaling pathways. IL-4, IL-13, TGF*β* and IL-1*β* could activate IMCs and enable their suppressive functions through the activation of STAT1, STAT6 and NF-*κ*B signaling pathways.^5^ Targeting MDSCs to reverse immunosuppressive tumor microenvironment is feasible and able to enhance the efficacy of cancer vaccines. All-*trans* retinoid acid and CpG-ODN could induce the differentiation of MDSCs into dendritic cells and macrophages *in vitro* and *in vivo*,^6, 7^ and thereby enhance the efficacy of cancer immunotherapy.

There is evidence showing that distinct energy metabolic programs are used by different immune cells to support their unique differentiation and functions.^8^ Neutrophils tend to use aerobic glycolysis instead of oxidative phosphorylation (OXPHOS) to generate energy.^9, 10^ Upon stimulation through TLRs, dendritic cells and macrophages at rest state switch from OXPHOS to aerobic glycolysis and become activated.^11, 12^ Naïve and memory T cells use OXPHOS for major energy source, whereas effector T cells switch to aerobic glycolysis supporting their proliferation and effector functions.^13, 14, 15^

During tumor progression, IMCs behave like cancer cells, which undergo massive proliferation and accumulate in a huge number in cancer patients. Given that the number of MDSCs existing in tumor-bearing hosts can affect the efficiency of tumor immune surveillance and prognosis of the patient, in the present study, we investigated the accumulation of MDSCs in tumor-bearing hosts. By comparing the microarray data, we found that MDSCs from tumor-bearing mice exhibited higher glycolytic rate than their normal cell compartment did. Upregulation of glycolysis prevented excess reactive oxygen species (ROS) production by MDSCs and protected these cells from apoptosis. Moreover, we identified the glycolytic metabolite, phosphoenolpyruvate (PEP), as an important antioxidant agent able to prevent excess ROS production by MDSCs. These findings suggest that glycolytic metabolites have important roles in the modulation of fitness of MDSCs and contribute to their massive accumulation in tumor-bearing individuals.

## Results

### MDSCs accumulated in the lymphoid organs and periphery in the tumor-bearing hosts

**Figure 1:**
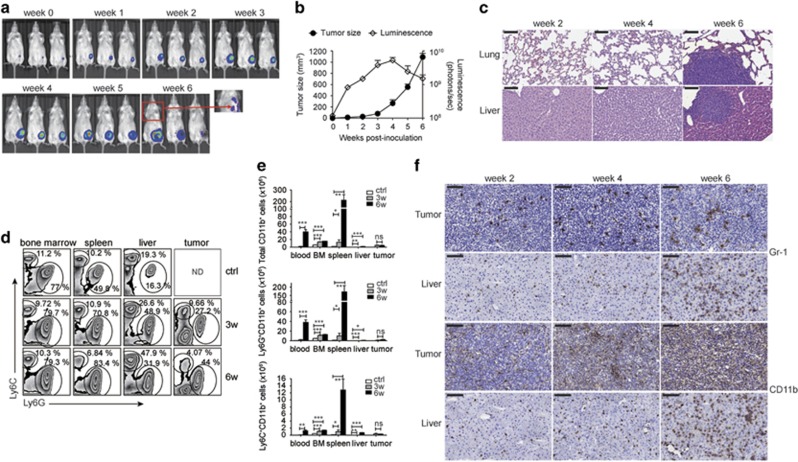
MDSC accumulation during tumor progression. (**a**) The IVIS images of Balb/c mice receiving 4T1-LG at indicated time points after implantation. Arrow indicated the IVIS image of the 4T1 lung metastasis in mice. (**b**) The tumor growth curve and total flux of luciferase activity of mice in (**a**) (*n*=3 per group). (**c**) The hematoxylin and eosin (H&E) staining of lung and liver sections of mice at indicated time points after inoculation of 4T1-LG tumor cells. (**d**) Representative flow cytometric analysis of Ly6G^+^ and Ly6C^+^ populations among gated CD11b^+^ cells in indicated tissues of control and 4T1-tumor-bearing mice at the third week and sixth week after tumor inoculation. (**e**) Absolute numbers of total CD11b^+^ cells, Ly6G^+^CD11b^+^ cells and Ly6C^+^CD11b^+^ cells in indicated organs (*n*=3 per group). (**f**) Immunohistochemical staining for detection of Gr-1^+^ and CD11b^+^ cells, respectively, in the liver and tumor sites of 4T1-tumor-bearing mice at indicated time points after tumor inoculation. All the data are representative of two independent experiments. ND, not done; ns, not significant. *P<0.05, **P<0.01, ***P<0.001 (unpaired Student’s *t*-test). (**c** and **f**) Scale bars, 100 *μ*m. (**b** and **e**) Error bars, S.D.

MDSCs had important roles in the progression of breast cancer and could serve as a prognostic biomarker for the disease stage or burden.^16^ We, therefore, investigated the induction and maintenance of MDSCs during cancer progression in an imageable 4T1 breast cancer mouse model generated through transduction of the parental 4T1 cell line with a plasmid expressing firefly luciferase and enhanced green fluorescence protein (eGFP) followed by puromycin selection. After inoculation of 2 × 10^5^ 4T1-LG cells into the mammary gland fat pad of female BALB/c mice, the *in vivo* bioluminescence was detected by *in vivo* imaging system (IVIS) (Figure 1a) and both the intensity of bioluminescence and size of the tumor increased gradually during the first 4 weeks (Figures 1a and b). Metastases to the lung and to the liver were observed at sixth week through detection of bioluminescence and microscopic metastases shown by tissue staining with hematoxylin and eosin (Figures 1a and c). We further analyzed the cell number of total CD11b^+^ cells, granulocytic MDSCs (gMDSC: Ly6G^+^ CD11b^+^) and monocytic MDSCs (mMDSC: Ly6C^+^CD11b^+^) in the blood, bone marrow, spleen, liver and tumor at the third week and sixth week after 4T1-LG implantation. The cell number of CD11b^+^ cells or MDSCs in all the tissues increased markedly after tumor implantation in comparison with the number in normal BALB/c female mice (Figures 1d and e). The CD11b^+^ cells recovered from the tumor mass comprised not only MDSCs but also CD11b^+^Ly6C^−^F4/80^+^ tumor-associated macrophages, which appeared abundantly in the primary tumor at both the third week and sixth week after inoculation (Supplementary Figure 1). The immunohistochemical staining of livers and tumor from the tumor-bearing mice from different time points using anti-Gr-1 (detecting MDSCs) and anti-CD11b (detecting all myeloid cells) antibodies also confirmed the accumulation of pathological myeloid cells in both sites (Figure 1f) during tumor progression.

### MDSCs from tumor-bearing mice upregulated glycolysis

**Figure 2:**
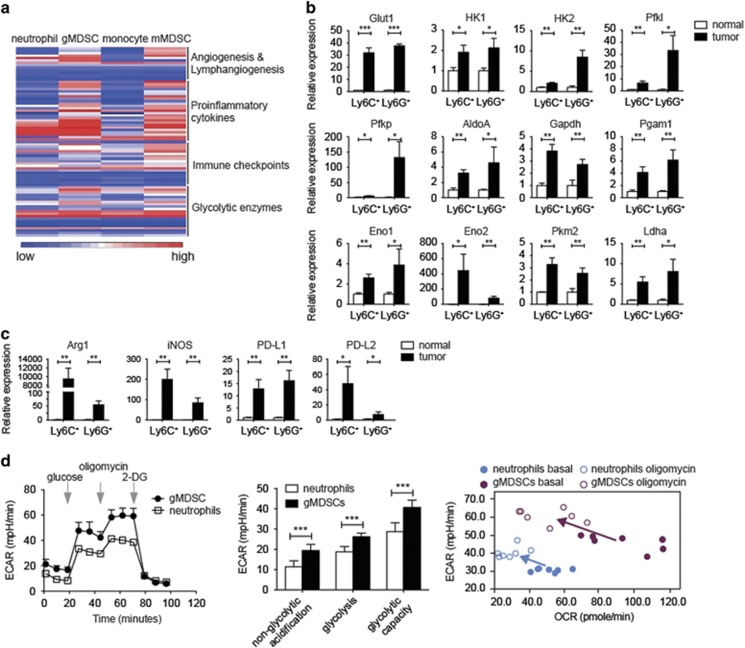
Metabolic status in myeloid cells during tumor progression. (**a**) Heat map summarizing the gene expression data from cDNA microarray. mRNA levels of genes of interest in splenic neutrophils, monocytes from normal healthy mice, gMDSCs and mMDSCs from tumor site of 4T1-tumor-bearing mice were shown. (**b**) Quantitative real-time PCR analysis of gene expression of glycolytic enzymes in FACSorted CD11b^+^Ly6C^+^ and CD11b^+^Ly6G^+^ cells from tumor sites of 4T1-tumor-bearing mice and spleens from normal BALB/c mice (*n*=3 per group). (**c**) Quantitative real-time PCR analysis of *ARG1*, *NOS2*, *PDCD1LG1* and *PDCD1LG2* in FACSorted CD11b^+^Ly6C^+^ and CD11b^+^Ly6G^+^ cells from tumor sites of 4T1-tumor-bearing mice and spleens from normal BALB/c mice (*n*=3 per group). (**d**) ECAR of FACSorted splenic gMDSCs from 4T1-tumor-bearing mice and splenic neutrophils from normal BALB/c mice (left panel); calculated non-glycolytic acidification, glycolysis and glycolytic capacity of the cells (middle panel); ECAR *versus* OCR of the above cells under basal condition and oligomycin treatment (right panel, *n*=7 per group). All the data are representative of two independent experiments. *P<0.05, **P<0.01 and ***P<0.001 (unpaired Student’s *t*-test). (**b**–**d**) Error bars, S.D.

For elucidation of the mechanisms mediating the massive accumulation of MDSCs during tumor progression, we compared the gene expression profiles of pathological and normal myeloid cells. Tumoral mMDSCs, gMDSC from 4T1-tumor-bearing mice, splenic neutrophils and monocytes from normal BALB/c mice were isolated for cDNA microarray analysis. The gene expression of angiogenesis/lymphangiogenesis factors, proinflammatory cytokines and immunosuppressive molecules/immune checkpoints increased in mMDSCs and gMDSCs in comparison with their normal cell compartments. Interestingly, the gene expression of nearly all glycolytic enzymes in mMDSCs and gMDSCs was also upregulated (Figure 2a). This microarray result was further validated by quantitative real-time PCR. The mRNAs of glycolytic enzymes, especially glucose transporter, Glut-1, phosphofructokinase, Pfkl and Pfkp isoforms, and enolase 2 (Eno2), were more abundant in MDSCs in comparison with their normal cell compartments (Figure 2b). Immunosuppressive features of these MDSCs were confirmed by detection of high levels of mRNAs of immunosuppressive molecules (Figure 2c). Enough amounts of splenic gMDSCs but not mMDSCs were able to be isolated for the measurement of extracellular acidification rate (ECAR) and oxygen consumption rate (OCR) by seahorse analyzer to reveal their status of glucose metabolism. gMDSCs exhibited higher ECAR than normal neutrophils through the entire measurement (Figure 2d, left panel). We could find that the non-glycolytic acidification, glycolysis and glycolytic capacity of gMDSCs were higher than those of neutrophils (Figure 2d, middle panel), which echoed the elevated mRNA levels of glycolytic enzymes in MDSCs (Figure 2b). The rate of glucose uptake of MDSCs from the tumor site and the spleen of tumor-bearing mice was significantly higher than that of monocytes or neutrophils from the spleen of normal control mice (Supplementary Figure 2). In general, normal neutrophils showed a more quiescent phenotype for their energy production by glycolysis or OXPHOS, whereas gMDSCs were more metabolically active to produce energy through both pathways (Figure 2d, right panel). Blockade of mitochondrial ATP synthesis by oligomycin resulted in an increase of ECAR and a decrease in OCR in both neutrophils and gMDSCs and also revealed higher glycolytic capacity in gMDSCs than in neutrophils (Figure 2d, right panel).

### Cancer cells promoted glycolysis in myeloid cells

**Figure 3:**
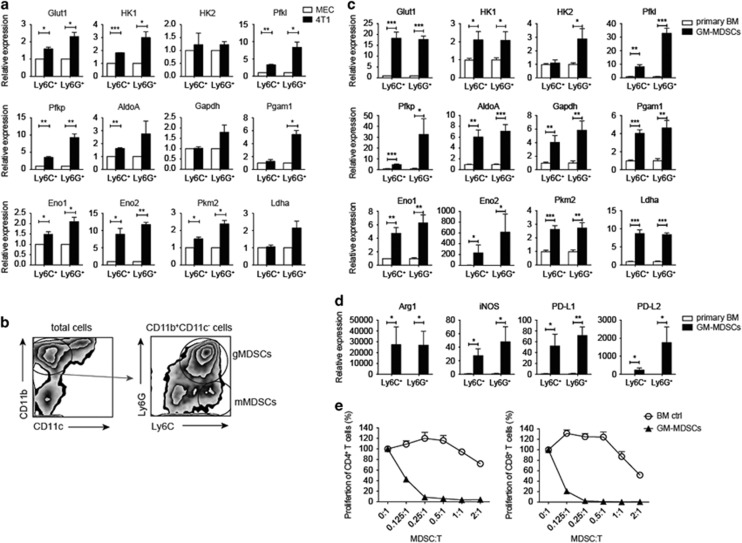
*In vitro* induction of MDSCs. (**a**) Quantitative real-time PCR analysis of gene expression of glycolytic enzymes in purified bone marrow neutrophils and monocytes after incubation with 4T1-tumor cells or with primary MECs for 40 h. (**b**) Representative flow cytometric zebra plots of MDSCs induced from bone marrow cells in the presence of GM-CSF for 5 days. CD11b and CD11c expression (left) of the total cells and Ly6C and Ly6G expression (right) of gated CD11b^+^CD11c^−^ cells were shown. (**c**) Quantitative real-time PCR analysis of gene expression of glycolytic enzymes in FACSorted GM-CSF-induced (GM-MDSCs) or bone marrow (primary BM) CD11b^+^CD11c^−^Ly6C^+^ and CD11b^+^CD11c^−^Ly6G^+^ cells (*n*=3 per group). (**d**) Quantitative real-time PCR analysis of *ARG1*, *NOS2*, *PDCD1LG1* and *PDCD1LG2* in the cells in (**c**) (*n*=3 per group). (**e**) Proliferation of CD4^+^ (left) and CD8^+^ (right) T cells cocultured with different numbers of MACSorted fresh bone marrow CD11b^+^ (BM ctrl) or MACSorted GM-CSF-induced CD11b^+^ MDSCs (GM-MDSCs). The percentage of the proliferating CD4^+^ (left panel) and CD8^+^ (right panel) T cells was calculated by dividing the number of EdU^+^ cells of each well by the average number of EdU^+^ cells in wells without MDSCs (as 100%). All the data are representative of two independent experiments. *P<0.05, **P<0.01 and ***P<0.001 (unpaired Student’s *t*-test). (**a** and **c**–**e**) Error bars, S.D.

We examined the influence of breast cancer cells on glycolysis of myeloid cells through coculture of bone marrow monocytes and neutrophils with 4T1 cells or primary mouse fat pad mammary epithelial cells (MECs) and found that both monocytes and neutrophils encountering 4T1 cancer cells showed higher levels of mRNAs of glycolytic enzymes, especially Pfkl, Pfkp and Eno2, in comparison with MEC-experienced cells (Figure 3a). GM-CSF alone or in combination with other cytokines was often used to induce MDSCs from bone marrow cells *in vitro*.^17, 18^ The levels of GM-CSF were found to increase in cancer patients, which induced accumulation of MDSCs.^19, 20, 21^ We therefore treated bone marrow cells from Balb/c mice with 20 ng/ml of GM-CSF for 5 days and found that more than 70% of Balb/c bone marrow CD11b^+^CD11c^−^ cells were Ly6G^hi^ gMDSCs and <10% of them were Ly6C^hi^ mMDSCs (Figure 3b). The ratio of the GM-CSF-induced mMDSCs and gMDSCs out from the bone marrow culture was similar to the ratio of them in the bone marrow and spleen of tumor-bearing mice in our study (Figure 1d) and in other breast cancer mouse model.^22^ FACSorted GM-CSF-induced gMDSCs and mMDSCs and bone marrow monocytes and neutrophils from Balb/c mice were then examined for their mRNA expression levels of glycolytic genes. Both GM-CSF-induced mMDSCs and gMDSCs expressed higher mRNA levels of glycolytic genes, especially *Pfkl*, *Pfkp* and *Eno2*, in comparison with their normal cell compartments (Figure 3c). The immune inhibitory characteristics of these GM-CSF-induced mMDSCs and gMDSCs were confirmed by their increased mRNA expression of Arg1, iNOS, PD-L1 and PD-L2 (Figure 3d), and their suppressive activity toward T-cell proliferation (Figure 3e).

### Blockade of glycolysis *in vivo* ameliorated MDSC accumulation and suppressed tumor progression

**Figure 4:**
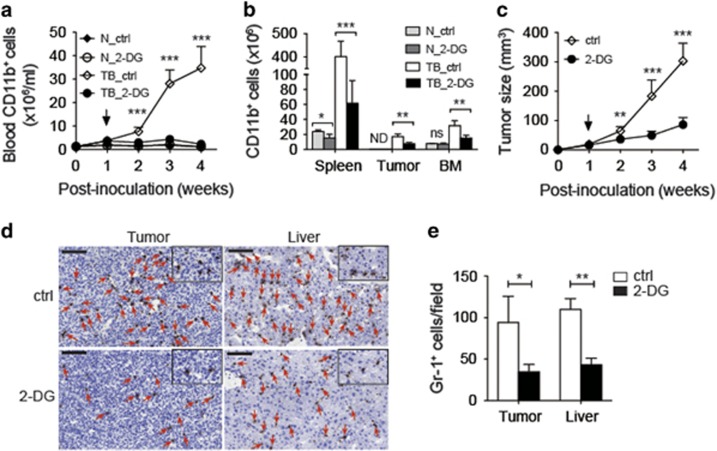
The *in vivo* accumulation of MDSCs was dependent on glycolysis. (**a**) Counts of CD11b^+^ cells in the blood of PBS-treated (ctrl) or 2-DG-treated normal (N) and 4T1-tumor-bearing (TB) mice at indicated time points after tumor inoculation. Arrow indicated the time point starting 2-DG treatment (*n*=3 per group). (**b**) Absolute numbers of CD11b^+^ cells in the indicated tissues of the mice in (**a**) at the fourth week after tumor inoculation (*n*=3 per group). (**c**) Size of tumor in the PBS-treated (ctrl) and 2-DG-treated tumor-bearing mice measured at the indicated time points (*n*=6–7 per group). (**d**) Immunohistochemical staining for detection and (**e**) quantification of Gr-1^+^ cells in the tumor and the liver of the PBS-treated (ctrl) and 2-DG-treated tumor-bearing mice at week 4 after tumor inoculation. Arrowheads indicated Gr-1^+^ cells (*n*=3 per group). Scale bars, 100 *μ*m. All the data are representative of two independent experiments. ND, not done; ns, not significant. *P<0.05, **P<0.01 and ***P<0.001 (unpaired Student’s *t*-test). (**a**–**c** and **e**) Error bars, S.D.

We further examined whether enhancement of glycolysis in myeloid cells contributed to the massive accumulation of MDSCs in tumor-bearing individuals. The tumor-bearing mice were intraperitoneally given 2-deoxy-d-glucose (2-DG), an inhibitor of hexokinase, at a dose of 1 g/kg body weight or DPBS (ctrl) every other day starting at second week after 4T1-tumor inoculation. Treatment of 2-DG for 1 week already significantly reduced the number of blood MDSCs in tumor-bearing mice (Figure 4a). A significant reduction of MDSCs in all examined tissues after 3 weeks of 2-DG treatment (Figure 4b) was observed. The 2-DG treatment at this dose for 3 weeks did not change the number of bone marrow myeloid cells but slightly reduced the number of splenic myeloid cells in normal mice (Figure 4b), which suggests that the maintenance of the number of normal myeloid cells does not rely on glycolysis as much as MDSCs do. Blockade of glycolysis significantly suppressed the tumor growth as revealed by the measurement of tumor size in Figure 4c. The number of Gr-1^+^ MDSCs decreased in the tumor and liver tissues of tumor-bearing mice after blockade of glycolysis (Figures 4d and e). However, it is difficult to distinguish the effect of 2-DG on tumor cells and on MDSCs in an *in vivo* tumor model, we therefore addressed the influence of glycolysis on MDSC expansion also in an *in vitro* culture system in later sections.

### The expansion of MDSCs was dependent on glycolysis

**Figure 5:**
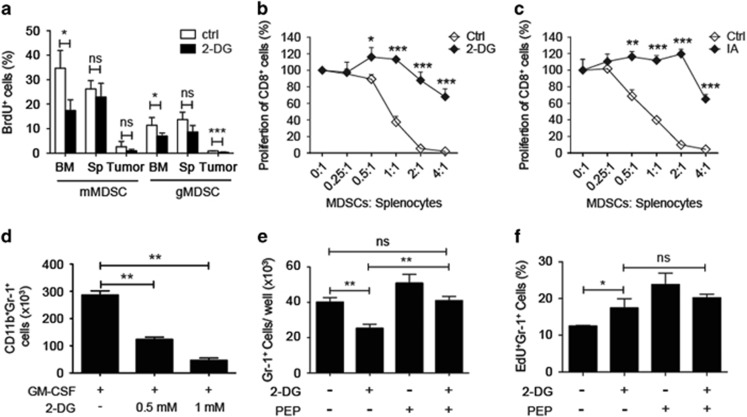
The expansion of MDSCs was dependent on glycolysis. (**a**) Percentage of BrdU^+^ cells among mMDSCs and gMDSCs from the bone marrow (BM), spleen (Sp) and tumor site of the PBS-treated (ctrl) and 2-DG-treated 4T1-tumor-bearing mice (*n*=3 per group). (**b**) Proliferation of CD8^+^ T cells cocultured with different numbers of splenic PBS (ctrl)-, 2-DG-treated CD11b^+^ MDSCs from 4T1-tumor-bearing mice. (**c**) Proliferation of CD8^+^ T cells cocultured with different numbers of splenic PBS (ctrl)-, IA-treated CD11b^+^ MDSCs from 4T1-tumor-bearing mice. (**d**) Cell numbers of CD11b^+^Gr-1^+^ MDSCs recovered from the MDSC induction culture 5 days after induction using 20 ng/ml of GM-CSF in the presence or absence of 2-DG in 24-well plates. (**e**) Cell numbers of CD11b^+^Gr-1^+^ MDSCs recovered from the MDSC induction culture 3 days after induction using 20 ng/ml of GM-CSF in the presence or absence of 0.5 mM 2-DG and PEP (5 mM) in 96-well plates. (**f**) Percentage of proliferating CD11b^+^Gr-1^+^ MDSCs among the cells from (**e**). All the data are representative of two independent experiments. ns, not significant. *P<0.05, **P<0.01 and ***P<0.001 (unpaired Student’s *t*-test). (**a**–**f**) Error bars, S.D.

Presuming that glycolysis facilitates the expansion of myeloid cell population in tumor-bearing individuals, it is important to clarify whether glycolysis promotes the proliferation of MDSCs or prevents their apoptosis. We treated the mice with 2-DG and analyzed the proliferative rate of the MDSCs. After blockade of glycolysis, the percentage of mMDSCs and gMDSCs incorporating BrdU, especially in the bone marrow of tumor-bearing mice, decreased significantly in comparison with that of control tumor-bearing mice (Figure 5a). The splenic CD11b^+^ MDSCs from tumor-bearing mice were isolated, treated with inhibitors of glycolysis (Supplementary Figure 3A) *ex vivo* and then added to splenocytes from Balb/c mice stimulated with anti-CD3/CD28 antibodies. Both 2-DG- and sodium iodoacetate (IA)-, an inhibitor of GAPDH, treated MDSCs showed less T-cell suppression activity (Figures 5b and c). Since we observed that *in vivo* blockade of glycolysis in tumor-bearing mice significantly suppressed the proliferation of myeloid cells in bone marrow but not in spleens of tumor-bearing mice, we postulated that the differentiation of bone marrow myeloid cell precursors into MDSCs in response to tumor-related factors was highly dependent on glycolysis. We then cultured bone marrow cells from healthy BALB/c mice in the presence of GM-CSF and 2-DG for 5 days and noticed that blockade of glycolysis significantly decreased the cell number of Gr-1^+^ MDSCs in comparison with the cell number recovered from control culture (Figure 5d). Blockade of glycolysis through inhibition of GAPDH by IA reduced the cell number of MDSCs (Supplementary Figure 3B), whereas inhibition of lactate dehydrogenase (LDHA) by oxamate did not significantly alter the cell number (Supplementary Figure 3C).

Inhibition of hexokinase and GAPDH but not LDHA could reduce cell expansion of MDSCs and Eno2 able to convert 2-phosphoglycerate into PEP was one of the highly unregulated glycolytic enzymes in MDSCs (Figure 2b). We therefore postulated that the metabolites downstream of GAPDH but upstream of LDHA, for example, PEP or pyruvate, may have important roles in the maintenance of MDSCs. However, abundant sodium pyruvate was provided (up to 1 mM) in the MDSC culture medium during blockade of glycolysis by 2-DG or IA. We therefore excluded pyruvate as the candidate glycolytic metabolite able to regulate survival of MDSCs. We then examined whether PEP was required for the GM-CSF-induced expansion of MDSCs. PEP was added to the MDSC induction culture in the presence of GM-CSF in combination with 2-DG or not. The cell numbers and percentage of proliferating Gr-1^+^ cells were analyzed at day 3. We found that PEP could rescue the 2-DG-induced suppression of MDSC expansion (Figure 5e). The proliferation of Gr-1^+^ cells during day 2 to day 3 was measured by 5-ethynyl-2′-deoxyuridine (EdU) incorporation followed by FACS analysis. To our surprise, blockade of glycolysis by 2-DG did not abolish the proliferation of the remaining Gr-1^+^ cells and even slightly increased the percentage of proliferating cells and the supplement of PEP during the blockade of glycolysis did not alter the percentage of proliferating Gr-1^+^ cells (Figure 5f), which suggests that blockade of glycolysis did not induce proliferation arrest of MDSCs during MDSC differentiation. This finding implies that upregulation of glycolysis in MDSCs could prevent them from apoptosis and the glycolytic metabolite, PEP, has a key role in the process of antiapoptosis of MDSCs.

### Glycolysis prevented excess ROS production and apoptosis of MDSCs

**Figure 6:**
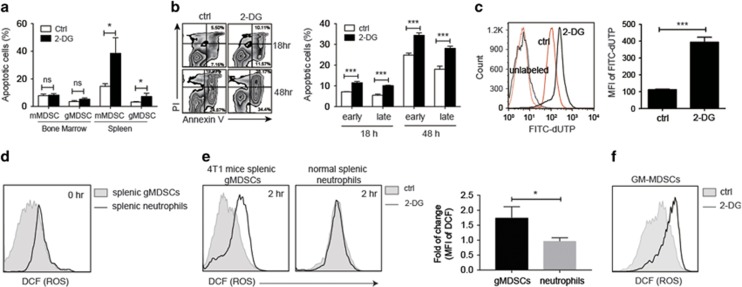
Glycolysis prevented apoptosis and excess ROS production of MDSCs. (**a**) Percentage of PI^−^Annexin V^+^ early apoptotic cells among mMDSCs and gMDSCs from the bone marrow and spleen of the PBS-treated (ctrl) and 2-DG-treated 4T1-tumor-bearing mice. 4T1-tumor-bearing mice were treated with 40 mg of 2-DG or PBS (ctrl) as described in Materials and Methods (*n*=3 per group). (**b**) Representative flow cytometric zebra plots gating on Gr-1^+^ population of *in vitro* 4T1-induced myeloid cells subjected to 2-DG or control treatment for 18 or 48 h showed PI and Annexin V staining. The bar graph of percentage of early (PI^−^Annexin V^+^) and late (PI^+^ Annexin V^+^) apoptotic cells among Gr-1^+^ population of *in vitro* 4T1-induced myeloid cells subjected to 2-DG or PBS (ctrl) treatment for 18 or 48 h was shown (*n*=3 per group). (**c**) Histogram (left) and bar graph (right) showing fluorescence intensity or mean fluorescence intensity (MFI) of FITC-dUTP-labeled or -unlabeled DNA fragments in MACSorted CD11b^+^Gr-1^+^ GM-CSF-induced MDSCs treated with 2-DG or PBS (ctrl) for 22 h and subjected to detection of DNA fragment by TUNEL assay (*n*=3 per group). (**d**) ROS production in freshly isolated neutrophils from normal BALB/c mice and in freshly isolated gMDSCs from 4T1-tumor-bearing mice. (**e**) Representative histograms (left two panels) of ROS production in neutrophils from normal BALB/c mice and in gMDSCs from 4T1-tumor-bearing mice treated with 2-DG or vehicle (DPBS, ctrl) for 2 h. Bar graph (right panel) showing the fold changes of MFI of DCFDA of the cells after treatment with 2-DG for 2 h. The MFI of DCFDA of the cells treated with vehicle was used as denominator (*n*=3 per group). (**f**) A representative histogram for ROS production in MACSorted GM-CSF-induced CD11b^+^Gr-1^+^ MDSCs treated with or without 10 mM of 2-DG for 6 h. All the data are representative of two independent experiments. ns, not significant. *P<0.05, **P<0.01 and ***P<0.001 (unpaired Student’s *t*-test). (**a–c** and **e**) Error bars, S.D.

We then examined whether glycolysis had a central role in the prevention of apoptosis of MDSCs and treated the tumor-bearing mice with 2-DG or DPBS (ctrl). The blockade of glycolysis in MDSCs by 2-DG significantly promoted apoptosis of splenic but did not affect that of bone marrow mMDSCs and gMDSCs (Figure 6a), which suggests that the MDSCs in the periphery are more dependent on glycolysis to prevent their apoptosis. We also examined the apoptotic rate induced by blockade of glycolysis of *in vitro*-induced MDSCs, which were bone marrow myeloid cells exposed to 4T1-tumor cells for 2 days. We found that these *in vitro*-induced Gr-1^+^ MDSCs were also susceptible to the blockade of glycolysis and increased both their early and late apoptotic rates (Figure 6b). We also measured the apoptosis of GM-CSF-induced MDSCs treated with 2-DG for 24 h through the detection of DNA fragmentation by TUNEL assay and found that the GM-CSF-induced MDSCs were also susceptible to blockade of glycolysis and showed a higher degree of DNA fragmentation in comparison with ctrl-treated MDSCs (Figure 6c). Blockade of glycolysis through inhibition of GAPDH by IA also induced apoptosis of them (Supplementary Figure 3D), whereas inhibition of LDHA by oxamate did not significantly alter the apoptotic rate of MDSCs (Supplementary Figure 3E).

ROS has been shown to be one of the inducers for oxidative stress, which could mediate apoptosis.^23^ We therefore measured ROS production of 2-DG-treated and ctrl-treated gMDSCs from both *in vivo* and *in vitro* by chloromethyl-H_2_DCFDA staining and compared that with the ROS production of normal splenic neutrophils under the same treatment. The ROS level of freshly isolated neutrophils was higher than freshly isolated gMDSCs (Figure 6d). *Ex vivo* blockade of glycolysis by 2-DG for 2 h did not induce an increase of ROS production in splenic neutrophils from normal Balb/c mice but enhanced ROS production in gMDSCs from 4T1-tumor-bearing mice (Figure 6e). The ROS level of gMDSCs after blockade of glycolysis reached the level similar to that of primary neutrophils (Figure 6e). GM-CSF-induced Gr-1^+^ MDSCs were also susceptible to blockade of glycolysis and showed higher ROS production in comparison with ctrl-treated MDSCs (Figure 6f).

### PEP-protected MDSCs from apoptosis through prevention of excess ROS production

**Figure 7:**
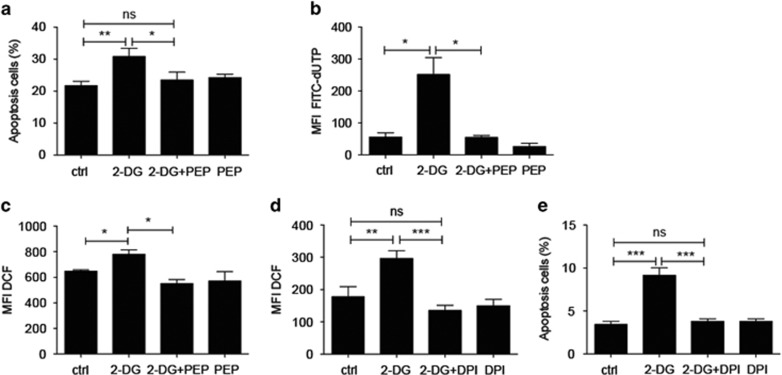
PEP ameliorated apoptosis in MDSCs through prevention of excess ROS production during blockade of glycolysis. (**a**) Percentage of PI^−^Annexin V^+^ early apoptotic cells among CD11b^+^Gr-1^+^ GM-CSF-induced MDSCs treated with 10 mM of 2-DG or vehicle (ctrl) for 8 h in the presence or absence of PEP at the concentration of 5 mM (*n*=3 per group). (**b**) MFI of FITC-dUTP-labeled DNA fragments in MACSorted Gr-1^+^ GM-CSF-induced MDSCs treated with 10 mM of 2-DG or vehicle (ctrl) for 22 h in the presence or absence of PEP at the concentration of 7.5 mM (*n*=3 per group). (**c**) ROS production in CD11b^+^Gr-1^+^ GM-CSF-induced MDSCs treated with 10 mM of 2-DG or vehicle (ctrl) for 5 h in the presence or absence of PEP at the concentration of 5 mM (*n*=3 per group). (**d**) ROS production in CD11b^+^Gr-1^+^ GM-CSF-induced MDSCs pretreated with 2 *μ*M of DPI or vehicle for 1 h before the addition of 2-DG at the concentration of 10 mM for additional incubation for 5 h (*n*=3 per group). (**e**) Percentage of PI^−^Annexin V^+^ early apoptotic cells among CD11b^+^Gr-1^+^ GM-CSF-induced MDSCs treated as in (**d**) (*n*=3 per group). All the data are representative of two independent experiments. ns, not significant. *P<0.05, **P<0.01 and ***P<0.001 (unpaired Student’s *t*-test). (**a–e**) Error bars, S.D.

PEP was found in our previous experiments to be able to rescue the cell expansion of Gr-1^+^ MDSCs suppressed by 2-DG during MDSC induction by GM-CSF (Figure 5e). We therefore examined whether PEP could rescue the 2-DG-induced apoptosis in MDSCs. We treated the GM-CSF-induced MDSCs with PEP during the blockade of glycolysis by 2-DG and analyzed the percentage of early apoptotic cells or detected the DNA fragmentation. PEP supplementation during the blockade of glycolysis significantly rescued the 2-DG-induced apoptosis (Figures 7a and b). ROS production of MDSCs was elevated during blockade of glycolysis (Figures 6e and f). We further examined whether PEP could prevent the production of ROS induced by the blockade of glycolysis, which subsequently precludes MDSCs from apoptosis. Indeed, PEP significantly prevented 2-DG-induced ROS elevation (Figure 7c). Treatment of NADPH oxidase inhibitor, diphenyleneiodonium (DPI), could significantly reduce ROS production by MDSCs (Figure 7d) and decreased the percentage of early apoptotic Gr-1^+^ MDSCs during the blockade of glycolysis (Figure 7e). These data demonstrated that the glycolytic metabolite, PEP, has an antioxidative capacity and could prevent apoptosis of MDSCs under oxidative stress.

## Discussion

Neutrophils have only a few mitochondria and therefore are shown to highly depend on glycolysis for ATP production.^24^ In this study, we compared the utilization of glycolysis by normal neutrophils, monocytes and pathological MDSC populations from tumor-bearing mice and found that both mMDSCs and gMDSCs upregulated their glycolysis in comparison with their normal cell compartments. We further demonstrated that upon direct interaction with cancer cells, or stimulation with GM-CSF, the bone marrow myeloid precursor cells would also switch to high utilization of glycolysis and developed their suppressive capabilities. It has been shown that aberration of glucose metabolism would result in neutrophil ER stress, oxidative stress and apoptosis, which caused neutropenia observed in glycogen storage disease-Ib- and G6Pase-*β*-deficient patients.^25, 26, 27, 28^ In this study, we demonstrated that upregulation of glycolysis in MDSCs contributed to cell survival of MDSCs and was highly related to the massive accumulation of MDSCs in tumor-bearing individuals. We observed that gMDSCs also used OXPHOS for energy production and therefore concluded that MDSCs are more metabolically active than neutrophils are. OXPHOS is a major cellular source of ROS. Cells with active OXPHOS require antioxidants to prevent oxidative stress and to keep cells fit. Our finding suggests that upregulation of glycolysis may help to keep the ROS level within a safe range and facilitate MDSC expansion.

The death of neutrophils and gMDSCs was shown to be highly regulated by both extrinsic and intrinsic (mitochondria) pathways of apoptosis.^24^ Signaling through death receptors, for example, Fas and TNF-related apoptosis-induced ligand receptors (TRAIL-Rs) on neutrophils or MDSCs, in the extrinsic pathway could trigger their apoptosis.^29, 30, 31^ Neutrophils and MDSCs both are able to produce a significant amount of ROS through the activation of NADPH oxidase for eliminating pathogens or mediating immunosuppression.^32, 33^ However, high ROS levels could also limit the lifespan of neutrophil through induction of intrinsic signaling leading to apoptosis.^34, 35^ We have found that neutrophils from normal mice expressed a higher level of ROS in comparison with that of gMDSCs from tumor-bearing mice. Blockade of glycolysis could induce additional ROS production in gMDSCs but not in neutrophils. Blockade of glycolysis in MDSCs rapidly induced ROS-mediated apoptosis, which suggests that MDSCs but not neutrophils may rely on high glycolytic rate to reduce ROS level, which prevents their apoptosis and enhances their accumulation during pathological conditions such as tumor progression and chronic inflammation.

PEP is a metabolite in the glycolysis pathway. In a previous study, PEP was shown to significantly attenuate the injury, oxidative stress and ATP depletion in livers during cold preservation for organ transplantation.^36^ This glycolytic intermediate was shown to accumulate in GM-CSF-induced MDSCs (data not shown) and prevent ROS production and apoptosis of MDSCs during the blockade of glycolysis in our studies. This result strongly suggests that the upregulation of glycolysis is a way for MDSCs to accumulate enough PEP for prevention of excess ROS production and maintenance of their survival. However, there is still little known about how PEP mediates antioxidant effect in cells. A possible explanation would be that PEP reduces ROS production through induction of ROS scavengers, for example, reduced glutathione, thioredoxin and NADPH or through induction of enzymes able to catalyze redox, for example, catalase, thioredoxin reductase and glutathione reductase, which requires further investigation.

## Materials and methods

### Reagents

2-DG (Merck Millipore, Darmstadt, Germany), mouse GM-CSF (BioLegend, San Diego, CA, USA), phosphoenolpyruvate (PEP) monosodium salt hydrate (Sigma-Aldrich, Saint Louis, MO, USA), sodium IA (Sigma-Aldrich), sodium oxamate (Sigma-Aldrich), DPI (Cayman, Ann Arbor, MI, USA).

### Animal studies

All mice at 5–7 weeks of age were purchased from the National Laboratory Animal Center (Taipei, Taiwan) and were kept in the laboratory animal center of NHRI. The two animal facilities are accredited by Association for Assessment and Accreditation of Laboratory Animal Care International (AAALAC International). The mice were maintained and treated according to the animal protocol (NHRI-IACUC-104005) approved by NHRI-IACUC. 4T1-tumor-bearing mice were treated with 2-DG at a dose of 40 mg per mouse or phosphate-buffered saline (PBS) as a control by intraperitoneal injection three times a week after tumor inoculation until the end of experiment. For the experiment of BrdU labeling *in vivo*, 4T1-tumor-bearing mice were treated with 2-DG three times every other day starting at the fourth week after tumor inoculation. One microgram of BrdU was given by intraperitoneal injection 6 h before killing. BrdU staining was performed using the BrdU Flow Kit (BD Biosciences, San Jose, CA, USA) according to the manufacturer’s instructions .

### Tumor model

An imageable 4T1-tumor cell line, 4T1-Luc-eGFP (4T1-LG), was established through transduction of 4T1-tumor cells with a lentiviral vector pLAS5W.puro (obtained from RNAi Core; Academia Sinica, Taipei, Taiwan) containing eGFP and luciferase open reading frames linked with 2A linker sequence. Recombinant lentiviruses were produced by co-transfection with viral vector pLAS5W-Luc-2A-eGFP, pCMV-ΔR8.91 and pMD.G in 293 cells according to the manufacturer’s instruction (RNAi Core; Academia Sinica). A total of 2 × 10^5^ 4T1-LG tumor cells was inoculated into the fourth mammary fat pad of 6–8-week-old female BALB/c mice. Tumor growth was monitored by bioluminescent imaging and physical measurement using calipers. The photons emitted from 4T1-LG cells within the live animals were detected and quantified using the IVIS (Caliper Life Sciences, Hopkinton, MA, USA). The tumor-bearing mice were injected intraperitoneally with 3 mg of d-luciferin (Biosynth Chemistry and Biology, Staad, Switzerland) and waited for 10 min before being imaged under anesthesia by isoflurane inhalation. Tumor volumes were calculated using the formula *V*=1/2(length x width^2^).

### Quantitative real-time PCR

Total RNA from cultured cells or sorted cells was purified by TRIzol reagent (Thermo Fisher Scientific, Waltham, MA, USA) and reversely transcribed into cDNA using SuperScript VILO cDNA Synthesis Kit (Thermo Fisher Scientific). KAPA SYBR FAST qPCR Kit (Kapa Biosystems, Wilmington, MA, USA) and corresponding primer sets (Supplementary Table 1) were used for quantitative real-time PCR analysis with Bio-Rad CFX96 Real-time System (Bio-Rad, Hercules, CA, USA). Relative expression was normalized to *β*-actin and calculated using 2^−ΔΔCt^ methods.^37^

### DNA microarray

Total RNA from FACSorted cells were purified using TRIzol reagent and RNA quality was checked by Bioanalyzer 2100 (Agilent Technologies, Santa Clara, CA, USA). DNA microarray analyses were performed using Affymetrix Mouse Gene 2.0 ST arrays by Microarray Core Laboratory of NHRI. mRNA levels of genes related to angiogenesis and lymphangiogenesis, proinflammatory cytokines, immune checkpoints and aerobic glycolysis in splenic neutrophils, monocytes from normal healthy mice and gMDSCs and mMDSCs from the tumor site of 4T1-tumor-bearing mice at the sixth week after tumor inoculation were shown in the heat map. RMA value (robust multiple-array average) from 2 to 14 (red color, higher expression; blue color, lower expression) was shown. The heat map was generated by the Partek software (Partek Inc., Saint Louis, MO, USA) with two-way data clustering.

### Purification of MDSCs, bone marrow myeloid cells and MECs

Tumor masses from tumor-bearing mice were minced into small pieces, digested in RPMI-1640 containing 0.5 mg/ml collagenase IA (Sigma-Aldrich) at 37 °C for 20 min and subjected to gradient centrifugation using Ficoll-Paque PLUS (GE Healthcare Life Sciences, Chicago, IL, USA) for isolation of leukocytes. Spleens were mechanically disrupted and treated with RBC lysis buffer to remove RBCs. Bone marrow cells in femurs were flushed out by DPBS. The total myeloid cells were purified using mouse CD11b^+^ Selection Kit (StemCell Technologies, Vancouver, BC, Canada). Bone marrow monocyte and mMDSCs of tumor-bearing mice were enriched using mouse monocyte Enrichment Kit (StemCell Technologies). Bone marrow neutrophil and gMDSCs of tumor-bearing mice were enriched using Mouse Neutrophil Enrichment Kit (StemCell Technologies). Gr-1^+^ GM-CSF-induced MDSCs were purified using APC-conjugated anti-Gr-1 (RB6-8C5) and APC^+^ Selection Kit (StemCell Technologies).

The isolation of MECs was performed according to the protocol described previously.^38^ Briefly, fourth and fifth fat pad from 8–12-week-old female mice were peeled away from the skin and minced. The mammary gland tissue was digested with 200 U/ml collagenase NB (Serva, Heidelberg, Germany) and 1% Pronase (Merck Millipore) at 37 °C for 30 min. After digestion, samples were filtered through metal mesh and centrifuged at 250 × *g* for 5 min at 4 °C. Then, the homogenate was resuspended in Trypsin-EDTA for further dissociation for 2 min at 37 °C and treated with 5 *μ*g/ml DNase I (Sigma-Aldrich) for another 5 min at 37 °C. Fetal bovine serum (FBS) was added to stop the reaction. The resultant cells were washed once with medium and resuspended in DMEM medium with 10% FBS into T75 flask and incubate for 2 h at 37 °C for fibroblast attachment. The non-attached cells were harvested and pellet down at the speed of 250 × *g* for 5 min. The resultant MECs were cultured in DMEM/F12 medium supplemented with 5% FBS, EGF (10 ng/ml), hydrocortisone (0.5 *μ*g/ml), bovine pituitary extract (50 *μ*g/ml) (Thermo Fisher Scientific) and 0.1% ITS premix (5 ng/ml insulin, 5 ng/ml transferrin and 5 pg/ml selenious acid) (Corning, Corning, NY, USA).

### Coculture of myeloid cells and tumor cells and MECs

For the gene expression analysis of MDSCs differentiation induced by 4T1-tumor cells *in vitro*, monocyte and neutrophil were isolated from bone marrow and enriched by Mouse Monocyte and Neutrophil Enrichment Kits (StemCell Technologies), respectively. Myeloid cells were cocultured with 4T1 cells at 2:1 ratio in 6-well plates. After 40 h of coculture, myeloid cells were isolated by mouse CD11b^+^ Selection Kit (StemCell Technologies). The purity of isolated CD11b^+^ cells achieved 95%. The harvested MDSCs were subjected to RNA extraction followed by reverse transcription and real-time PCR.

### Induction of MDSCs *in vitro*

Bone marrow cells were treated with GM-CSF (20 ng/ml) for 5 days at 1 × 10^6^ cells per ml in RPMI medium supplemented with 10% FBS, 25 mM  l-glutamine, 5 mM HEPES, 1 mM sodium pyruvate and 50 *μ*M *β*-mercaptoethanol. After 3- day induction, the culture medium with GM-CSF was refreshed and the cells were cultured for another 2 days before analysis.

### Immunohistochemical staining

Five-*μ*m section of frozen liver or tumor tissues were fixed in ice-cold acetone for 6 min. Gr-1 (RB6-8C5) and CD11b (M1/70) were purchased from BioLegend. The staining was performed using ImmPRESS Anti-Rat Ig, Mouse Adsorbed (peroxidase) Polymer Detection Kit, DAB Peroxidase Substrate Kit and Hematoxylin (Vector Laboratories, Burlingame, CA, USA). The slides were mounted with VectaMount Permanent (Vector Laboratories). The image was captured using an automatic Digital Slide Scanner Pannoramic MIDI with Plan-Apochromat 20x/0.8 objective (3D HISTECH, 3DHISTECH Ltd., Budapest, Hungary) by Pathology Core Laboratory of NHRI.

### Flow cytometry analysis

For quantification of myeloid cells in blood, 30 *μ*l of blood were collected into RBC lysis buffer on ice by tail bleeding before surface marker staining and flow cytometric analysis. The cell number was calculated as × 10^6^/ml by using CountBright Absolute Counting Beads (Thermo Fisher Scientific). Bone marrow cell number was shown as × 10^6^/mice from two femurs. The cell number of tumor, spleen and liver were adjusted to weight and expressed as × 10^6^/g.

All stainings for flow cytometric analysis were performed in the presence of 10 *μ*g/ml of Fc block (2.4G2) in fluorescence-activated cell sorting buffer (FACS buffer, PBS/2% bovine serum albumin/0.02%NaN_3_). Acquisition and data analysis were conducted on a FACSCalibur or LSR II (BD Biosciences) and FlowJo Software (V.10.0.8r1; FlowJo, LLC, Ashland, OR, USA). Antibodies and dye used for flow cytometric analysis were purchased from BD Biosciences, eBioscience, BioLegend or Thermo Fisher Scientific and were as following: FITC or PE-conjugated anti-CD11b (M1/70), PE-Cy7 or APC-conjugated anti-Ly6C (HK1.4), PerCp-Cy5.5-conjugated anti-F4/80 (BM8), PE-Cy7 or APC-conjugated anti-Ly6G (1A8), APC or PerCp-eFluor660-conjugated anti-Gr-1 (RB6-8C5) and FITC-conjugated anti-CD11c (N418), 7-AAD and LIVE/Dead Fixable Near-IR Dead Cell Stain Kit (Thermo Fisher Scientific).

### Assays for glucose metabolism

ECAR as an indicator of aerobic glycolysis and OCR an indicator of oxidative phosphorylation were measured using Seahorse XF Glycolysis Stress Test Kit on XFe 24 Extracellular Flux Analyzer (Agilent Technologies) following the manufacturer’s instructions. ECAR of the myeloid cells were measured in XF media containing 2 mM  l-glutamine under basal condition and in the presence of 10 mM  l-glucose, 1 *μ*M oligomycin and 100 mM 2-DG.

### Apoptosis assay

Detection of apoptotic cells was performed using FITC Annexin V Apoptosis Detection Kit I (BD Biosciences) or APO-DIRECT Kit (BD Biosciences) followed by surface marker staining and flow cytometric analysis. The early apoptotic cells were defined as Annexin V-positive and PI-negative populations and the late apoptotic cells were defined as Annexin V-positive and PI-positive populations.

### ROS detection

Detection of ROS was performed using chloromethyl- H_2_DCFDA (Thermo Fisher Scientific) followed by surface marker staining and flow cytometric analysis.

### *In vitro* T-cell suppression assay

A total of 2x10^5^ splenocytes stimulated by 1 *μ*g/ml anti-CD3/anti-CD28 antibody were cocultured with various numbers of MDSCs for 48 h. The proliferation of T cells was measured by incorporation of EdU, a thymidine analog. EdU (4 *μ*M) was added at 6 h before harvest of cells. EdU staining was performed using Click-iT EdU Alexa Fluor 488 Flow Cytometry Kit (Thermo Fisher Scientific).

### *In vitro* MDSC proliferation assay

MDSC proliferation assay was performed on GM-CSF-induced MDSCs at day 3. The harvested MDSCs were treated with 2-DG and metabolites in the presence of 4 *μ*M EdU for 16 h. The staining of EdU labeling was performed using Click-iT Plus EdU Alexa Fluor 488 Flow Cytometry Kit (Thermo Fisher Scientific).

### Statistical analysis

GraphPad Prism 7 (GraphPad Software, La Jolla, CA, USA) and Student's *t*-test were used for statistical analysis.
