# Supplementary material for: Glycolysis regulates the expansion of myeloid-derived suppressor cells in tumor-bearing hosts through prevention of ROS-mediated apoptosis
Source: Cell Death Dis. 2017 May 11;8(5):e2779–. doi: 10.1038/cddis.2017.192 (PMC5520713; doi:10.1038/cddis.2017.192)
Supplement: Supplementary Information [file cddis2017192x1.pdf]

Target	Primer	Sequence 5´-> 3'	Amplification length (bp)	Annealing temp (^{o}C)
betaAct	FW	AGAGGGAAATCGTGCGTGAC	151	60
	RV	CAATAGTGACCTGGCCGT		
Arg1	FW	CTCCAAGCCAAAGTCCTTAGAG	185	60
	RV	AGGAGCTGTCATTAGGGACATC		
iNOS	FW	CTGTGTGCCTGGAGGTTCTG	180	60
	RV	CCAATCTCTGCCTATCCGTCTC		
PD-L1	FW	GCTCCAAAGGACTTGTACGTG	238	60
	RV	TGATCTGAAGGGCAGCATTTC		
PD-L2	FW	CTGCCGATACTGAACCTGAGC	126	61
	RV	GCGGTCAAAATCGCACTCC		
Glut1	FW	CAGTTCGGCTATAACACTGGTG	156	61
	RV	GCCCCCGACAGAGAAGATG		
Hk1	FW	CGGAATGGGGAGCCTTTGG	269	61
	RV	GCCTTCCTTATCCGTTTCAATGG		
Hk2	FW	TGATCGCCTGCTTATTCACGG	112	61
	RV	AACCGCCTAGAAATCTCCAGA		
Pfkl	FW	CGACCGAATCCTGAGTAGCA	186	60
	RV	GCCTCGTCAAACCTCTCCTC		
Pfkp	FW	AGTGTCTGGCGTCTCTACCT	151	60
	RV	CAGCAGCATTCATGCCTTGG		
AldoA	FW	CGTGTGAATCCCTGCATTGG	180	61
	RV	CAGCCCCTGGGTAGTTGTC		
Gapdh	FW	GCAGTGGCAAAGTGGAGATT	249	60
	RV	TCTCCATGGTGGTGAAGACA		
Pgam1	FW	GTTGCGAGATGCTGGCTATGA	102	60
	RV	CACATCTGGTCAATGGCATCC		
Eno1	FW	TGCGTCCACTGGCATCTAC	118	61
	RV	CAGAGCAGGCGCAATAGTTTTA		
Eno2	FW	GTCCCTGGCCGTGTGTAAG	200	61
	RV	CATCCCGAAAGCTCTCAGC		
Pkm2	FW	GCCGCCTGGACATTGACTC	145	61
	RV	CCATGAGAGAAATTCAGCCGAG		
Ldha	FW	TGTCTCCAGCAAAGACTACTGT	155	60
	RV	GACTGTACTTGACAATGTTGGGA		

Supplementary table 1 Primer sequences used in quantitative real-time PCR.

Supplementary figure 1

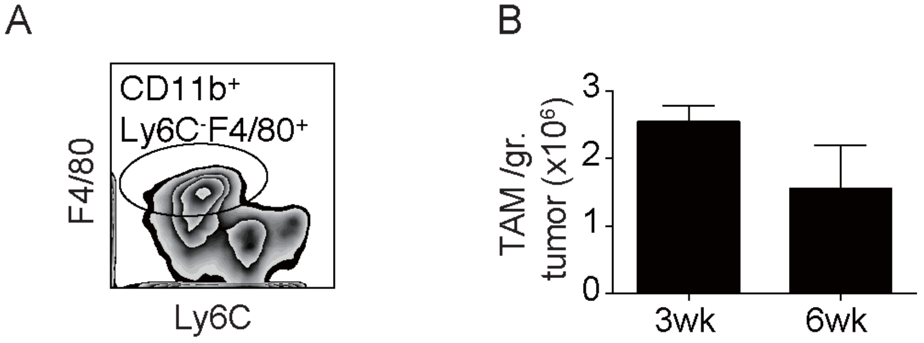

Supplementary figure 1 (**A**) A representative flow cytometric zebra plot (left) gating on total $CD11b^{+}$ population isolated from tumor site showing the F4/80 and Ly6C expression on the $CD11b^{+}$ cells and (**B**) quantification of cell number of tumor-associated macrophages (TAM, right) ($CD11b^{+}F4/80^{+}Ly6C^{-}$ population) in the tumor sites at indicated time points after tumor inoculation.

Supplementary figure 2

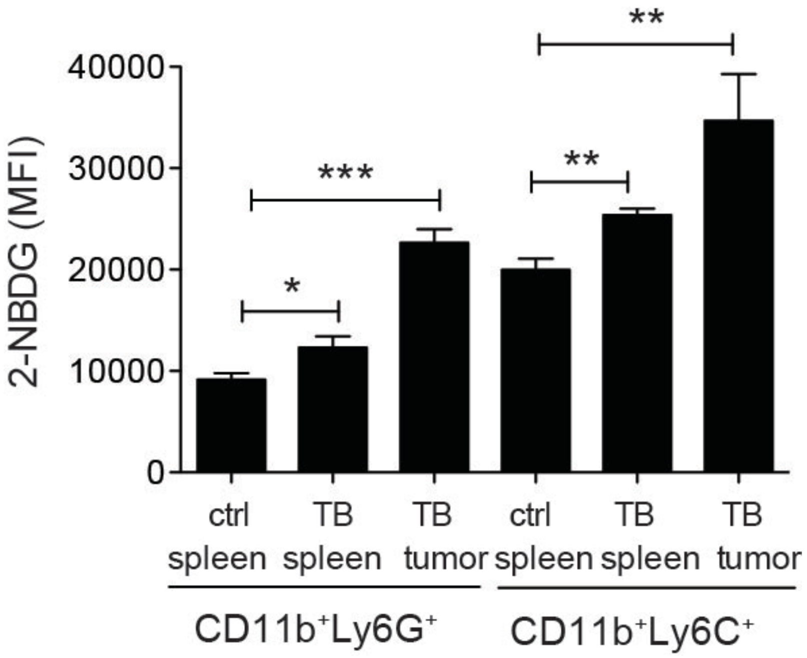

Supplementary figure 2 Glucose uptake of myeloid cells from the spleen of normal control (ctrl) mice and of MDSCs from the spleen and the tumor site of 4T1 tumor-bearing (TB) Balb/c mice. Splenocytes and tumor-associated leukocytes were cultured in glucose-free medium for 1 hour and pulsed with a fluorescent glucose analog - 2-NBDG (2-(*N*-(7-Nitrobenz-2-oxa-1,3-diazol-4-yl)Amino)-2-Deoxyglucose) at the concentration of 200 μM for another 1 hour. $CD11b^+Ly6G^+$ (gMDSCs, neutrophils) and $CD11b^+Ly6C^+$ (mMDSCs, monocytes) live cells were analyzed for their uptake of 2-NBDG by flow cytometry. *$P < 0.05$, **$P < 0.01$, ***$P < 0.001$ (unpaired Student's *t*-test). (error bars, s.d.)

Supplementary figure 3

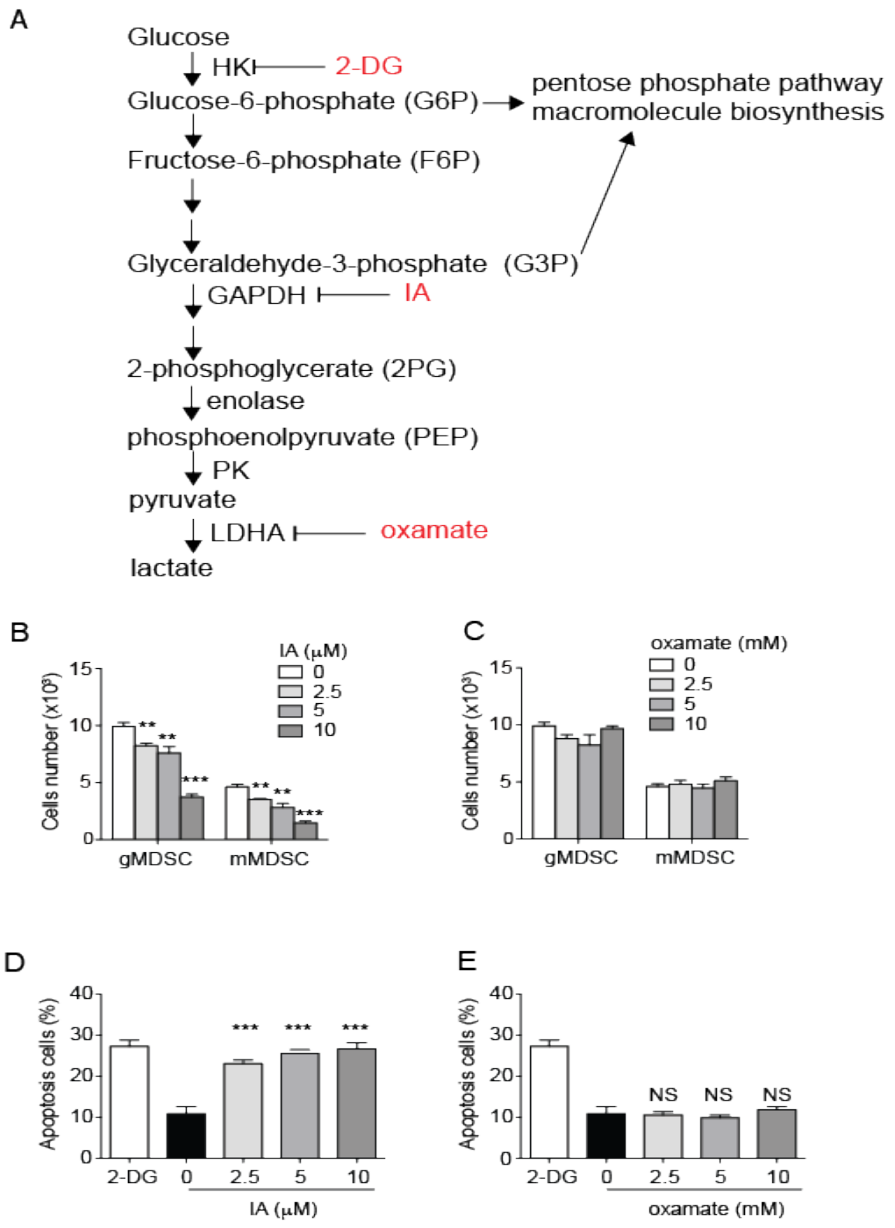

Supplementary figure 3 GAPDH inhibitor-IA but not LDHA inhibitor-oxamate also induced apoptosis of MDSCs. (**A**) Metabolites, enzymes and inhibitors (red) involved in aerobic glycolysis (**B**) Cell numbers of gMDSCs and mMDSCs recovered from the MDSC induction culture 3 days after induction using 20 ng/ml of GM-CSF in the presence or absence of indicated concentration of IA in 96-well plates. (**C**) Cell numbers of gMDSCs and mMDSCs recovered from the MDSC induction culture 3 days after induction using 20 ng/ml of GM-CSF in the presence or absence of indicated concentration of oxamate in 96-well plates. (**D**)

Percentage of $PI^-Annexin\ V^+$ early apoptotic cells among $CD11b^+Gr\text{-}1^+$ GM-CSF-induced MDSCs treated with IA for 8 hours. (**E**) Percentage of $PI^-Annexin\ V^+$ early apoptotic cells among $CD11b^+Gr\text{-}1^+$ GM-CSF-induced MDSCs treated with oxamate for 8 hours. NS, not significant., $*P < 0.05$, $**P < 0.01$, $***P < 0.001$ (unpaired Student's *t*-test). (**B, C, D, E**; error bars, s.d.)
